# Diffuse lesion and necrosis tied to poorer prognosis of interdigitating dendritic cell sarcoma: cases report and a pooled analysis

**DOI:** 10.1038/s41598-017-00719-2

**Published:** 2017-04-06

**Authors:** Feng Shi, Qingkun Song, Lingling Wang, Ying Gao, Hong Chang

**Affiliations:** 1 Department of pathology, Beijing Shijitan Hospital, Capital Medical University, Tieyi Road 10, Haidian District, Bejing 100038 China; 2 Department of science and technology, Beijing Shijitan Hospital, Capital Medical University, Tieyi Road 10, Haidian District, Bejing 100038 China

## Abstract

Interdigitating dendritic cell sarcoma is a neoplastic proliferation of interdigitating dendritic cells and no therapeutic consensus exists. This study aimed to investigate the prognostic impacts of tumor lesion, cellular atypia, mitosis and necrosis on the interdigitating dendritic cell sarcoma. Case reports and pooled analyses were designed to explore the relationships. One case was a 40-years old man with localized lesion, moderate to notable cellular atypia, 30 mitoses per 10 high-power fields and no necrosis and the progression-free survival was longer than 20 months. The other case was a 62-years old woman with diffuse lesion, notable cellular atypia, less than one mitosis per 10 high-power fields and diffuse necrosis and the progression-free survival was shorter than 1 month. Cellular atypia and mitosis had not any relationship with survival. Compared with localized lesion, diffuse lesion presented a 2.92-fold risk of progression (HR = 2.92, 95% CI 1.01, 8.51) and an 8.79-fold risk of death (HR = 8.79, 95% CI 1.86, 41.64). Diffuse necrosis presented a 4.39-fold higher progression risk (HR = 5.39, 95% CI 1.78, 16.29) and a 5.37-fold higher death risk (HR = 6.37, 95% CI 1.46, 27.86) than focal or no necrosis. Diffuse lesion and diffuse necrosis were indicators of poorer prognosis and the clinical application should be warranted in further studies.

## Introduction

Dendritic cells (DCs) are professional antigen-presenting cells that participate in both innate and adaptive immune response. They consist of some heterogeneous cells: Langerhans cells, dermal dendrocytes, follicular dendritic cells and interdigitating dendritic cells (IDC). IDC are non-lymphoid accessory cells, responsible for major histocompatibility complex restricted stimulations of resting T cells and localize in T cell zones of lymphoid organs, such as paracortex and deep cortex of lymph nodes, periarteriolar lymphoid sheaths in spleen and interfollicular areas of lymphoid tissue on mucosa^1, 2^. Interdigitating dendritic cell sarcoma (IDCS) is a neoplastic proliferation of heterogeneous IDC, such as spindle and ovoid cells. The phenotypic features were similar to IDC, positively expressing S100, weakly positive for CD68, lysozyme and CD45, but negative for CD21, CD23, CD35, CD1a and Langerin. IDCS is a very rare disease, with a challenging diagnosis. Under electron microscope, there were no signs of Birbeck granules^3, 4^. The clinical guidelines including the histopathology, prognostic factors and treatment protocol, have not yet confirmed. IDCS cases had a variation in prognosis: some cases had good outcomes, some cases worsened and even died, and some cases experienced another hematopoietic or solid organ malignancy^3, 5–14^. As to the heterogeneous clinical features, this study aimed to make two cases report and a pooled analysis of reported cases in order to clarify prognostic potency in histopathological factors and provide the practical reference of oncologist and pathologist.

## Result

### Pathological findings

#### Gross appearance

**Figure 1:**
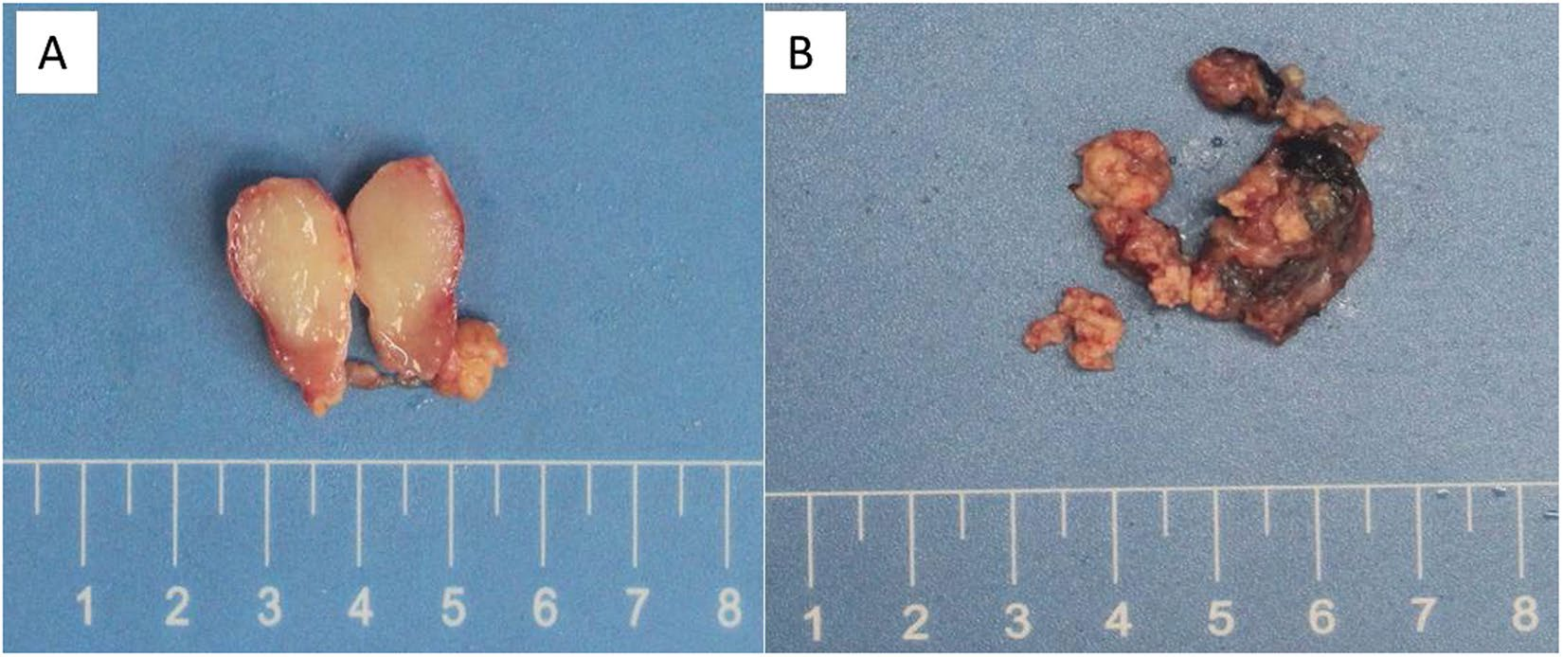
The section of case 1 was solid, white-grayish, fine and smooth, no bleeding and necrosis (**A**). Case 2 presented smashed white-grayish lymph nodes and the section was solid, white-grayish, focal black, rough and patchy necrosis (**B**).

The IDCS appeared to be a white-grayish solid tumor had some bleeding or necrosis. Case 1 presented a 1.3 × 1.0 × 0.8 cm black-grayish lymph node and the section showed solid, white-grayish, fine and smooth, no bleeding and no necrosis (Fig. 1A). Case 2 presented 4.0 × 2.5 × 1.0 cm smashed white-grayish lymph nodes and the section showed solid, white-grayish, focal black, rough and diffused necrosis (Fig. 1B).

#### Histopathology and immunochemistry

**Figure 2:**
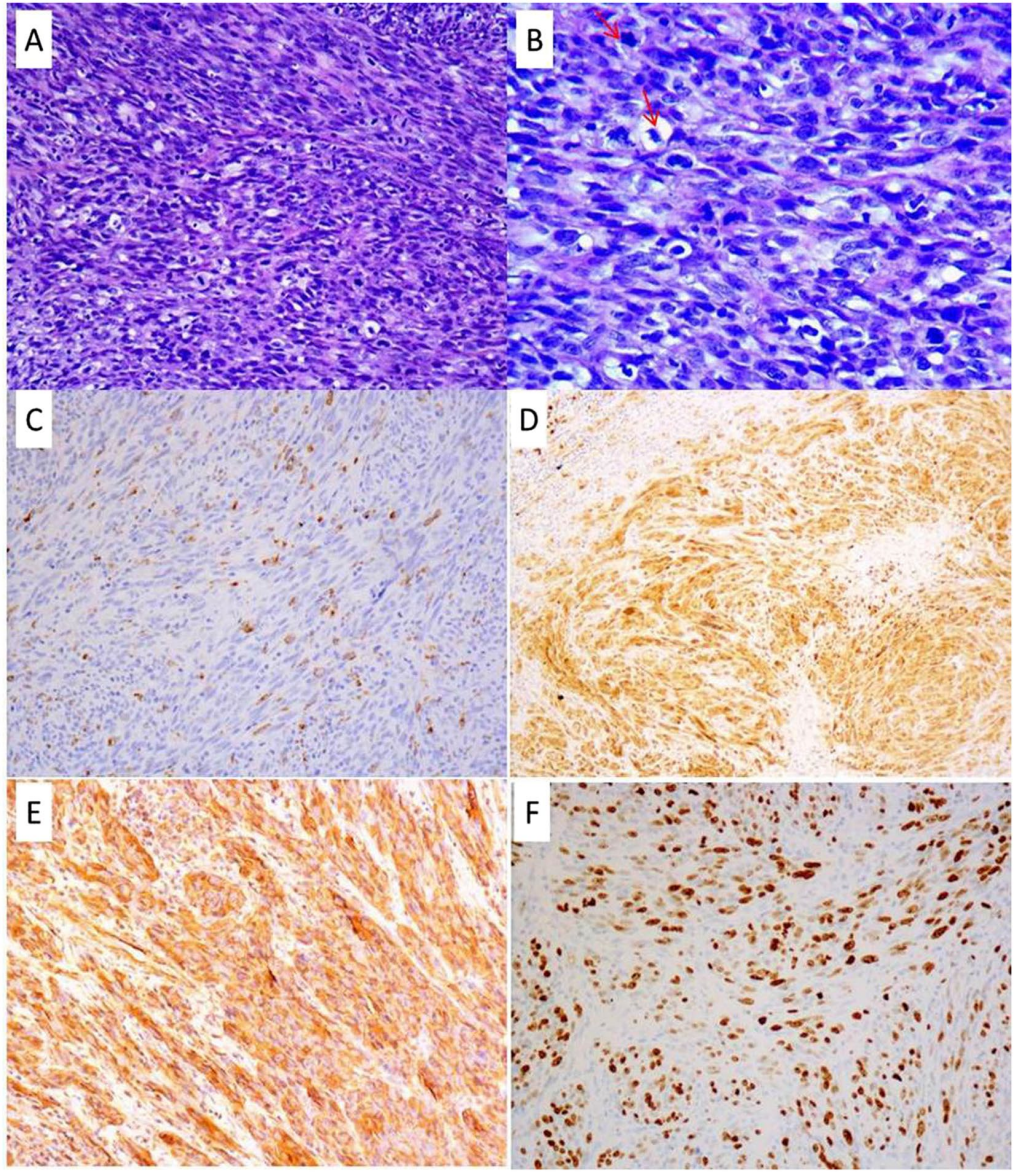
Histologic sections of case 1 displayed the spindle tumor cells disposing in fascicles with a focal storiform pattern (**A**). Neoplastic cells had irregular nuclei, moderately to prominent nucleoli, and notable mitoses (**B**). The tumor cells positive for CD68 (**C**), S100 (**D**), Vimentin (**E**) and Ki-67 index 80% (**F**).

Case 1: Histologic sections showed the lymph node replaced by spindle cells of moderate/severe cellular atypia. Neoplastic cells were disposed in fascicles and a focal storiform pattern. The nucleoli were irregular and presented bizarre, binucleated, or multinucleated forms, with 30 mitoses/10 high-power fields (HPF) (Fig. 2A,B). There was no necrosis. The tumor cells showed positive of CD68 (Fig. 2C), S100 (Fig. 2D), Vimentin (Fig. 2E) and Ki-67 index 80% (Fig. 2F). CKpan, cam5.2, EMA, CD21, CD23, CD35, SMA, HMB45, Melan A, CD1a, GFAP, ALK, LCA, CD117, CD34 and Myoglobin were negative in the cells.

**Figure 3:**
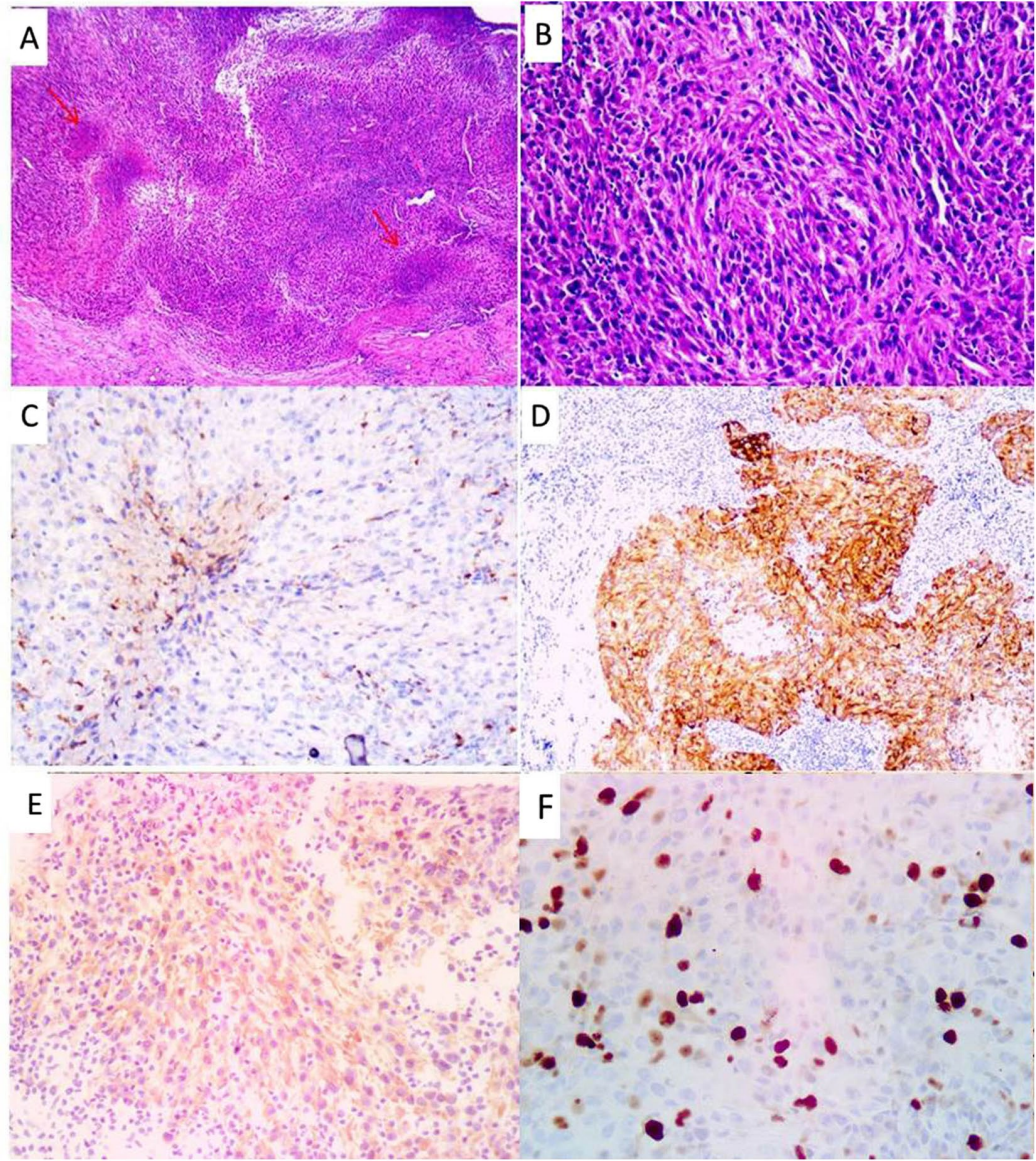
Histologic sections of case 2 displayed the sheets of atypia of epithelium-like cells and patchy necrosis (red arrow) (**A**). The tumor cells were spindle to oval, had abundant cytoplasm and prominentnucleoli (**B**). The tumor cells expressed CD68 (**C**), S100 (**D**), CKpan focal positive (**E**) and Ki-67 index 30% (**F**).

Case 2: Section showed the lymph nodes destroyed and replaced by epithelium-like cells of severe cellular atypia. The tumor cells included spindle and oval cells, had a diffuse distribution and showed abundant cytoplasm, prominent nucleoli and rare mitoses (less than 5/50 HPF) (Fig. 3A,B). The necrosis was diffuse. The tumor cells expressed CD68 (Fig. 3C), S100 (Fig. 3D), CKpan (Fig. 3E) and Ki-67 index 30% (Fig. 3F). Cam5.2, EMA, CD21, CD23, CD35, SMA, HMB45, Melan A, CD1a, GFAP, ALK, LCA, CD117, CD34 and Myoglobin were negative.

#### ISH and PCR

Detection of Epstein-Barrvirus–encoded small RNA was negative for two cases by *in situ* hybridization. T and B cell polymerase chain reaction (PCR) clonality studies were not rearrangement. Braf with V600E mutation was not detected.

#### Electron microscopic analysis

**Figure 4:**
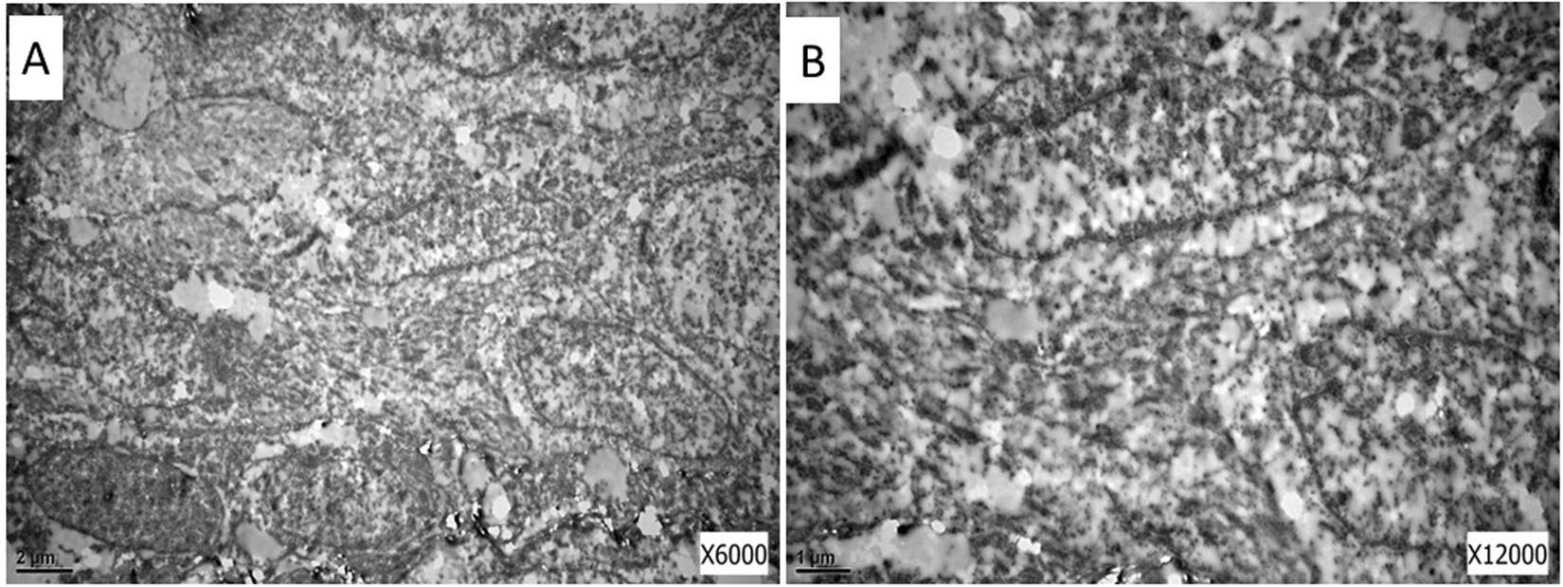
Electron microscopic sections presented the mutual interlaced long finger bumps and small amount of rough endoplasmic reticulum in cytoplasm.

The tumor cells had mutual interlaced long finger bumps and the borderline was unclear in the two cases. There were small amount of rough endoplasmic reticulum in cytoplasm. Nucleus had dents. There was no a typical bridge (Fig. 4A,B).

#### Treatment regimens and outcomes

Case 1 received three cycles of the CHOPE regimen, consisting of 1 g cyclophosphamide (day 1), 70 mg pirarubicin (day 1), 4 mg vindesine (day 1), 15 mg dexamethasone (days 1–5) and 100 mg etoposide (days 2–4), and six cycles of the DHAP regimen, consisting of 20 mg dexamethasone (days 1–4),100 mg cisplatin (day 1) and 500 mg cytarabine (Q12H days 2). Currently, the patient was followed upto 20 months, and no disease progression or distant metastasis occurred. The case 2 patient had two cycles of paclitaxel and nedaplatin regimen, but the disease was progressed.

#### Pooled analysis

**Figure 5:**
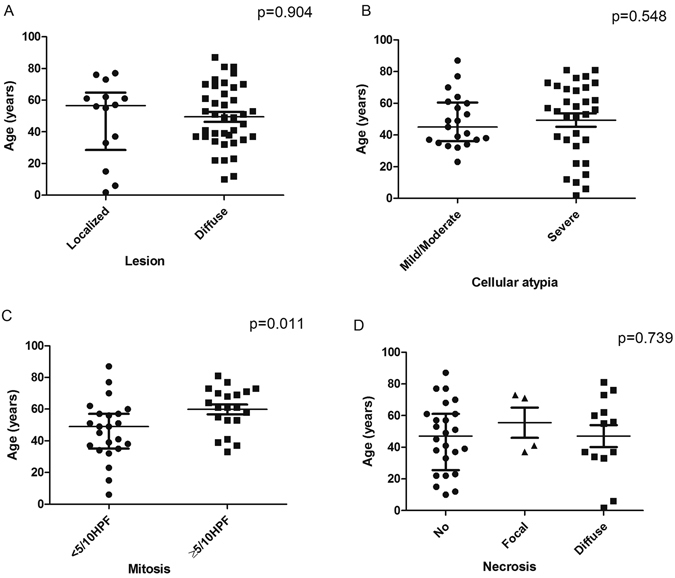
Median age has not any significant differences between tumor stage (**A**), nuclear abnormalities (**B**) and necrosis (**D**) (p > 0.05). Median age was 49 years old among patients with nuclear mitosis <5/10HPF, 12 years younger significantly than patients with nuclear mitosis ≥5/10HPF (**C**) (p = 0.011).

Median age of included cases was 51.0-year old, with interquartile range of 30.0 (Table 1). 47.2% cases were females, 26.4% cases had a localized lesion, 39.6% cases had mild/moderate cellular atypia, 46.5% cases had 5 mitoses per 10HPF at least and 31.7% cases had diffuse necrosis (Table 1). Median age was similar between histopathological factors of tumor lesion, cellular atypia and necrosis (p > 0.05) (Fig. 5A,B,D). However, median age was 49 years old among patients with nuclear mitosis less than 5/10HPF, 12 years younger than patients with nuclear mitosis equal or higher than 5/10HPF (p = 0.011) (Fig. 5C).

**Table 1:** General characteristics of included cases in the pooled analysis.

Items	Distribution
Age (Median, interquartile range)	51.0, 30.0
Gender, n (%)	
Male	25 (47.2)
Female	28 (52.8)
Lesion, n (%)	
Localized	14 (26.4)
Diffuse	39 (73.6)
Cellular atypia, n (%)	
Mild/moderate	21 (39.6)
Severe	32 (60.4)
Mitosis, n (%)	
<5/10HPF	23 (53.5)
≥5/10HPF	20 (46.5)
Necrosis, n (%)	
No	24 (58.5)
Localized	4 (9.8)
Diffuse	13 (31.7)

**Figure 6:**
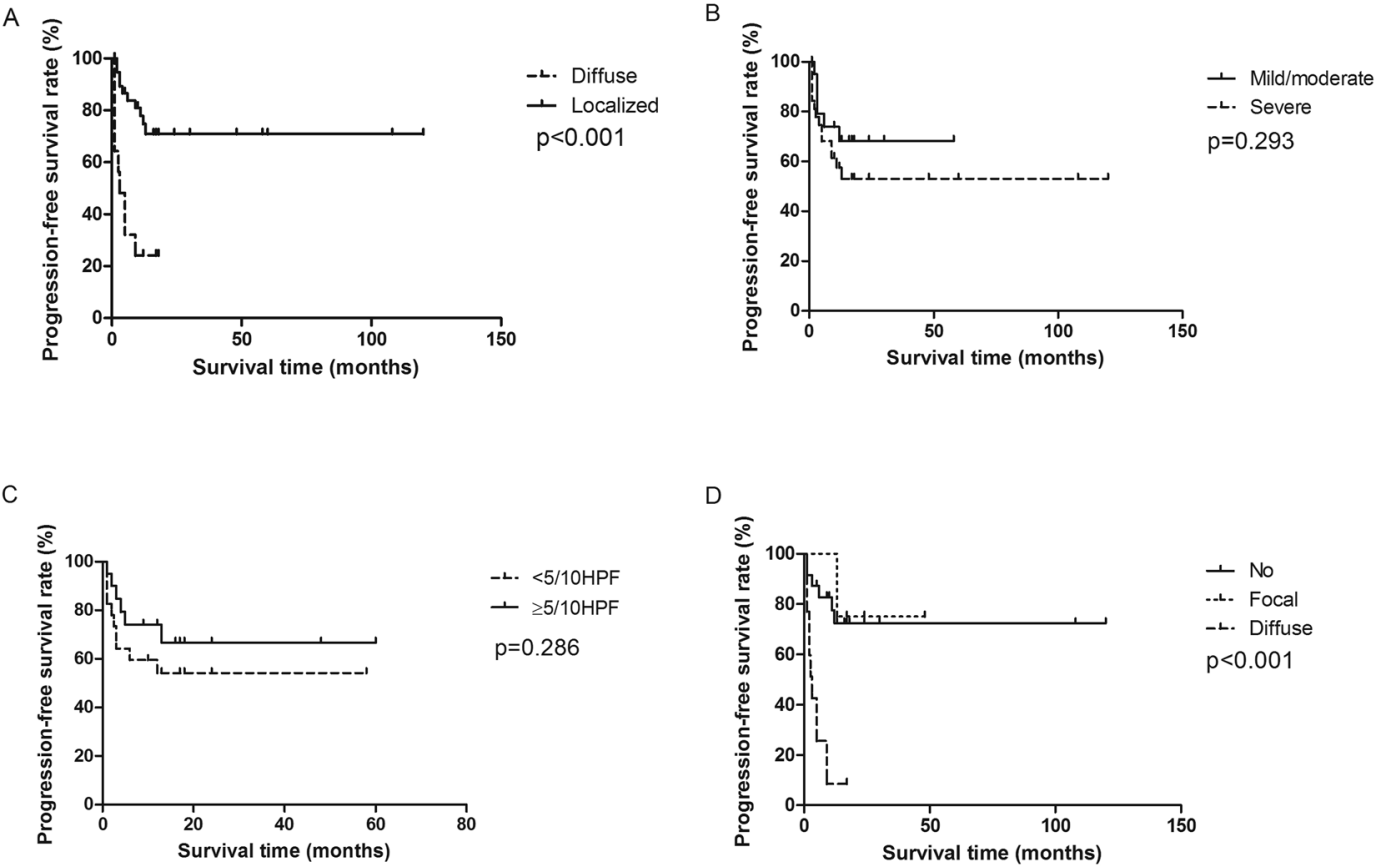
Patients with diffused stage had median PFS of 3 months, in contrast, patients with localized stage had not reached median PFS (**A**). Nuclear abnormalities and mitosis were not associated with PFS (**B**,**C**). Median PFS of diffuse necrosis was 3 months but patients with no necrosis or localized necrosis had not observed median PFS, with a significant difference (**D**).

Patients with diffused lesion had median PFS of 3 months, in contrast, patients with localized lesion had not reached median PFS (Fig. 6A). The difference was significant. Cellular atypia and mitosis were not associated with PFS (Fig. 6B,C). Median PFS of diffuse necrosis was 3 months but patients with no necrosis or focal necrosis had not observed median PFS, the difference being significant (Fig. 6D). In multivariate analysis, diffuse lesion presented a 2.92-fold risk of progression (HR = 2.92, 95% CI 1.01, 8.51) and an 8.79-fold risk of death (HR = 8.79, 95% CI 1.86, 41.64) (Table 2).

**Table 2:** Multivariate analysis between pathological characteristics and survival*.

	PFS			OS		
	HR	95% CI	p-value	HR	95% CI	p-value
Age	1.00	0.98, 1.02	0.729	1.00	0.97, 1.03	0.840
Gender						
Female	1			1		
Male	0.68	0.24, 1.95	0.470	0.56	0.14, 2.16	0.396
Lesion						
localized	1			1		
diffuse	2.93	1.01, 8.51	0.048	8.79	1.86, 41.64	0.006
Necrosis						
No/focal	1			1		
Diffuse	5.39	1.78, 16.29	0.003	6.37	1.46, 27.86	0.014

*COX regression model adjusting all factors above.

**Figure 7:**
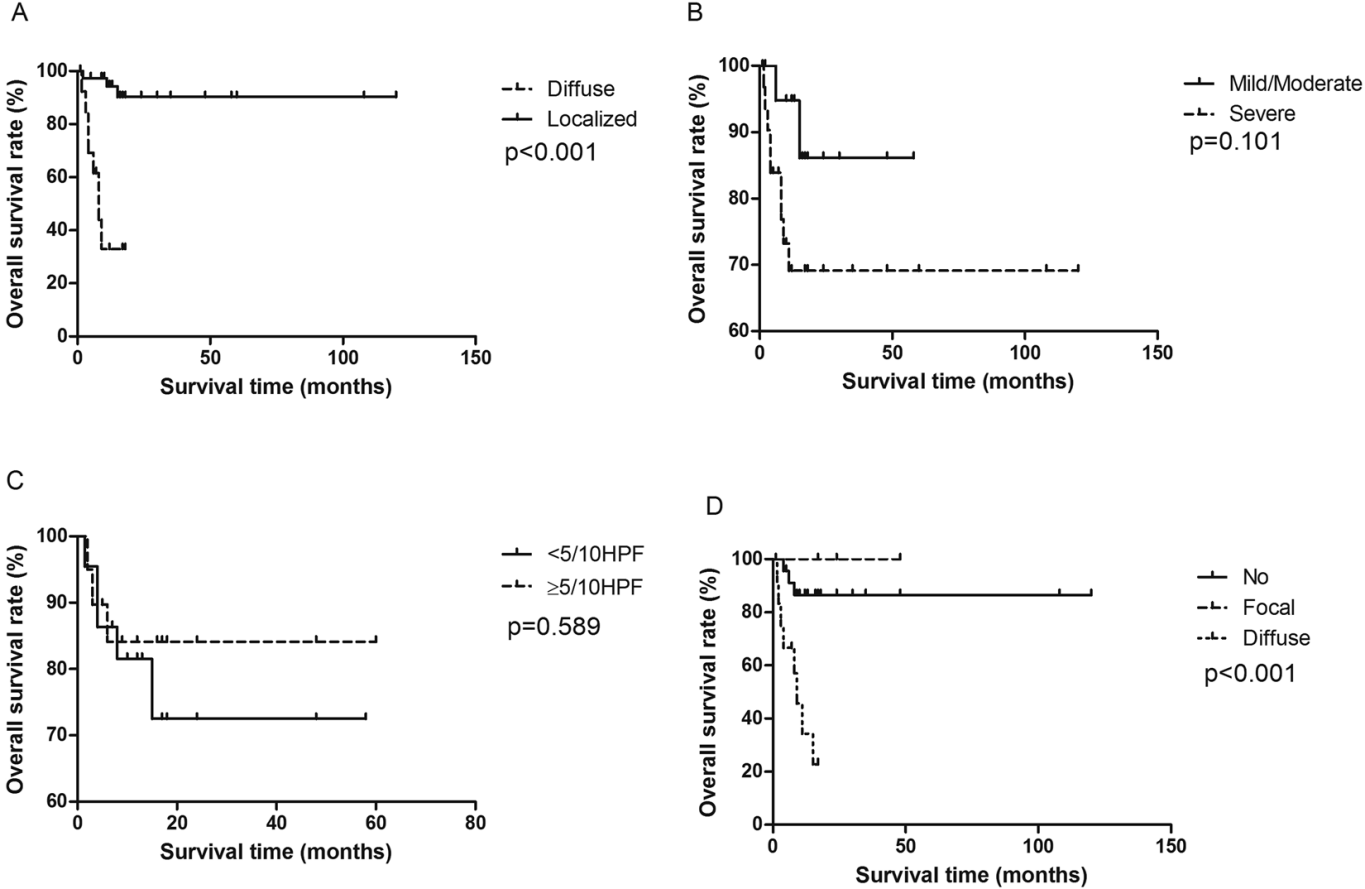
Tumor stage was associated with a shorter OS significantly (**A**). Nuclear abnormalities and mitosis were not related with OS (**B**,**C**). Patients with no or localized necrosis did not get median OS, compared with patients with diffused necrosis having a 9-month median OS (**D**) (p < 0.001).

Diffuse tumor lesion was associated with a shorter OS significantly (Fig. 7A). Patients with localized lesion did not reach median OS, but patients with diffuse lesion had a 9-month OS. Cellular atypia and mitosis were not related with OS (Fig. 7B,C). Patients with no or focal necrosis did not get median OS, compared with patients with diffused necrosis having a 9-month median OS (p < 0.001) (Fig. 7D). Diffuse necrosis was associated with a 5.39-fold high progression risk (HR = 5.39, 95% CI 1.78, 16.29) and a 6.37-fold high death risk (HR = 6.37, 95% CI 1.46, 27.86) (Table 2).

## Materials and Methods

This study was approved by Beijing Shijitan Hospital Institutional Review Board (IRB). Written informed consent was obtained from all patients. No identifying information or image was published. All of the methods were in accordance with the particular regulations.

### Study design

This study design consisted of a case report and a pooled analysis.

### Pooled analysis

This study chose the databases of PubMed and CHINAINFO. PubMed was accessed through NCBI and CHINAINFO was accessed through WANFANG DATA.

Search term was “interdigitating dendritic cell sarcoma”. The publication date ranged from Jan. 1, 1980 to Mar. 1, 2016. The language covered English and Chinese. Included article type was original research and the excluded types were reviews, meeting abstract, commentaries or re-analysis of data. All of the include IDCS cases should had pathlogical diagnosis in the publication. The endpoint outcome was progression-free survival (PFS), and overall survival (OS). The extracted data were age, gender, tumor lesion, cellular atypia, mitosis, necrosis, PFS and OS.

126 articles were obtained in PubMed and 53 articles were obtained in CHINAINFO. We excluded 42 articles in PubMed and 11 articles in CHINAINFO without IDCS cases. We reviewed all of the rest articles and found 34 cases in PubMed and 17 cases in CHINAINFO with histopathology data and outcome data.

Our two IDCS cases came from Beijing Shijitan hospital. The confirmed diagnosis covered by histological review of morphology in slides stained with hematoxylin and eosin, detection of a wide immunohistochemistry panel and application of electron microscope. The two cases were included in the pooled analysis.

### Case report

**Figure 8:**
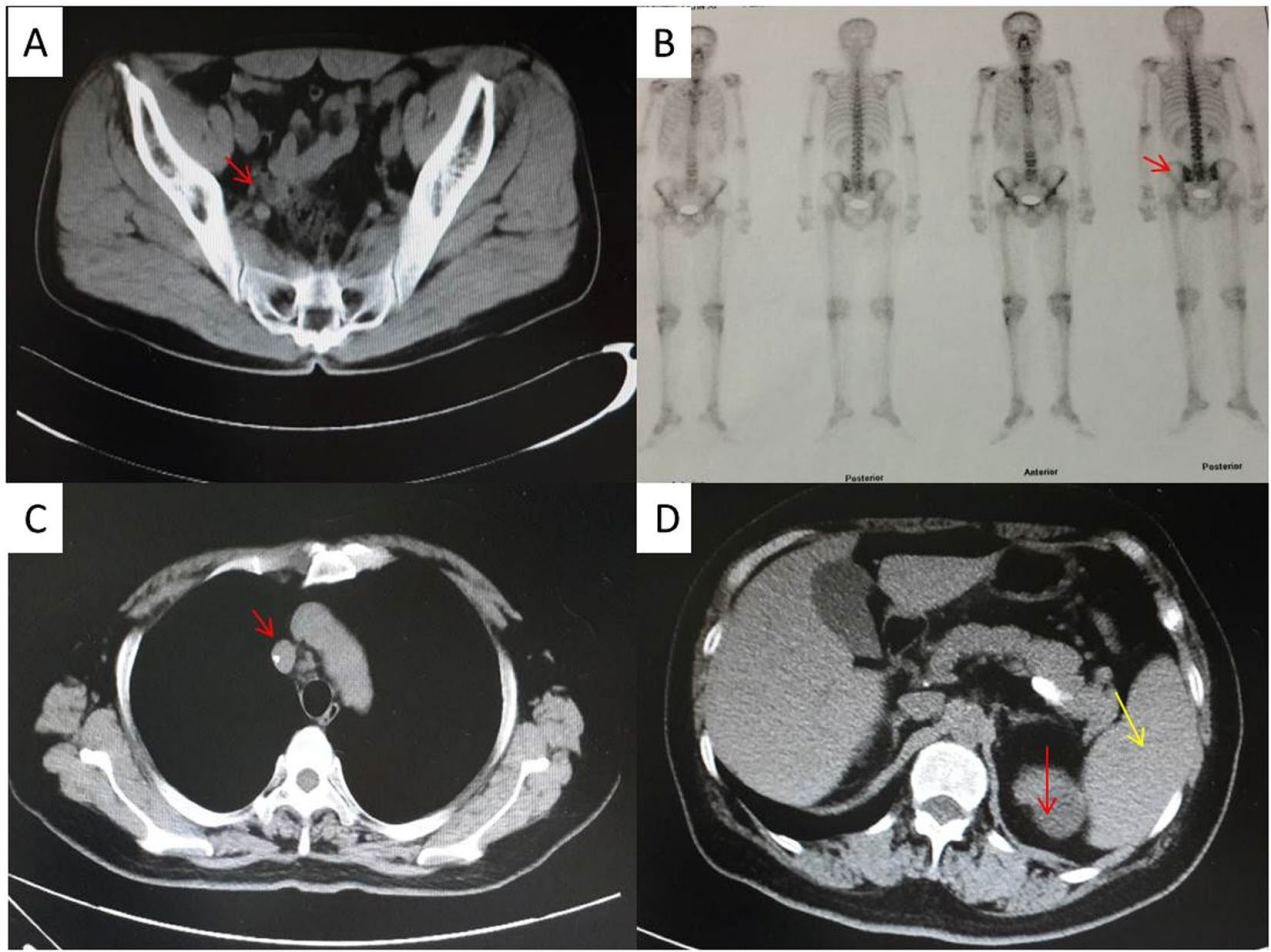
Pelvic CT scanning in case 1 displayed the lymph nodes aside the left external iliac blood vessels enlarged, with the biggest size of 1.8 × 1.4 cm (**A**). PETCT in case 1 showed a high bone salt metabolism in right coracoid and left sacroiliac joint (**B**). Chest CT scanning in case 2 displayed multiple lymph nodes enlarged in right hilar and mediastinal (**C**). Abdomen CT scanning in case 2 displayed a clear borderline mass in the right kidney (red arrow) and a slightly larger spleen (yellow arrow) (**D**).

Case 1: a 40-years old man visited the outpatient clinic presenting a two-week history of a painless mass in his left inguinal. The patient had foot nail removal for paronychia caused by trauma five years ago. No skin moles and tumor was found at lower limbs. The patient had no family cancer history and was in good health before. Ultrasound showed one low echo node in the right neck, the size being 0.9 × 0.2 cm; some low echo nodes in the left neck, the biggest size being 0.5 × 0.3 cm; some low echo nodes in the right inguinal, the biggest size being 0.8 × 0.3 cm; some low echo nodes in the left inguinal, the biggest size being 1.3 × 1.0 cm. All of the nodes had clearly border and regular contour. Pelvic CT (Fig. 8A) showed the lymph nodes aside the left external iliac blood vessels enlarged and the biggest one was 1.8 × 1.4 cm, but there was no enlarged lymph node in the right external iliac blood vessels and retroperitoneal. There was no abnormality in the liver and spleen. PETCT (Fig. 8B) displayed a high bone salt metabolism in right coracoid and left sacroiliac joint, indicating a benign lesion. Blood and bone marrow aspiration were normal.

Case 2: a 62-years old woman presented cough and hard breath for 3 months. Superficial lymph nodes were not touched and blood test was normal. Chest CT (Fig. 8C) scan showed: multiple lymph nodes enlarged in right hilar and mediastinal, multiple nodules with calcification in the upper lobe of right lung and some patchy enhancement spot in the middle of the upper lobe, left lower lobe and secondary adjacent bronchiectasis. Abdomen CT (Fig. 8D) scan showed a clear borderline mass in the right kidney, with the size of 4.4 × 4.1 × 5.8 cm, and a slightly larger spleen. Mediastinal lymph node was biopsied and pathological diagnosis was tending to be metastasis of poorly differentiated carcinoma with a large number of necrosis. The patient had two cycles chemotherapy of paclitaxel and nedaplatin but the disease progressed. The patient had mediastinal lymph node biopsy again.

### Definition

Diffuse lesions means the lesion involved other organs outside the primary site. Focal necrosis is small, usually no more than 0.3 mm, and the margin is relatively regular, cannot be seen in the gross appearance; diffuse necrosis is large, usually more than 0.5 mm, and has irregular margin, often be seen in the gross appearance, it is also been called “map-like” necrosis.

### Immunochemistry

All immunohistochemical stains were performed on the Ventana Benchmark automated staining system using 4 mm paraffin tissue sections. The primary antibodies used in this study including CD68, S100,CKpan, cam5.2, EMA, CD21, CD23, CD35, SMA, HMB45, Melan A, CD1a, GFAP, ALK, LCA, CD117, CD34, Myoglobin and Vimentin.

### Electron microscopic analysis

The tissue was postfixed in buffered osmium tetroxide, dehydrated in ethanol and embedded in Spurr’s resin. Ultrathin sections were prepared, stained with uranyl acetate–lead citrate and examined using a transmission electron microscope.

### ISH and PCR

*In-situ* hybridization was conducted through Epstein-Barr virus (EBV)-encoded small RNA assay (EBVit; catalogue number ISH-5021; OriGene Technologies, Inc., Rockville, MD, USA). Polymerase chain reaction (PCR) analysis was conducted with IdentiClone™ IGH GeneClonality Assay (catalogue number 9-101-0020; InvivoscribeTechnologies, Inc., San Diego, CA, USA). The PCR procedure started by denaturation at 95 °C for 7 min and followed by 35 cycles of 95 °C for 45 sec, 60 °C for 45 sec and 72 °C for 90 sec and a final extension at 72 °C for 10 min. BRAF V600E mutation analysis was performed to detect a T > A transversion of nucleotide 1799 of the BRAF oncogene. Tissue from the corresponding unstained slide was lysed, and the genomic DNA was purified from the paraffin block. Real-time PCR using a single primer set was used to amplify the region of the BRAF gene, which contained the mutation site, and two different fluorogenically labeled probes were used to detect the wild-type and V600E mutant.

### Statistical analysis

All analyses were conducted by SPSS 17.0 (IBM. Inc). The difference of age between tumor stage, nuclear abnormalities and nuclear mitosis was estimated by Mann Whitney test. Age difference between necrosis was analyzed by Kruskal-Wallis test. Kaplan-Meier survival curves were estimated for PFS and OS. Log-rank test was used to indicate the difference of survival curves. Age, gender, tumor stage and necrosis were analyzed together in COX Hazard Proportional Regression Model to present hazard ration (HR) and 95% confidence interval (95% CI). All tests were two-sided and the significant level was 0.05.

## Discussion

IDCS is an unusual variant of DCs tumor and regarded as a tumor of true hematopoietic origin^4^. DCs are specialized as professional antigen-presenting cells, which are important in the cell-mediated adaptive immune response to foreign antigens through the activation of T-cells. Within the lymph node, DCs can be classified into migratory DCs and resident DCs. Migratory DCs, such as Langerhans cells and dermal dendritic cells, are responsible for transporting antigens from distant sites to the lymph node for cross-presentation to T-cells. Resident DCs include follicular DCs and IDC. IDC reside in t-zones in the peripheral lymphoid tissues, such as the paracortex and deep cortex of lymph nodes. Dendritic neoplasms are rare hematological malignancies with less than 1% occurrence in the lymph nodes or soft tissues. IDCS can occur in lymph node, nasopharynx, small intestine, mesentery, spleen, testis, skin, tonsil, bladder, eyelid, uterine cervix and so on^6, 9, 13–15^. Usually, IDCS had an indistinct border, astoriform pattern of spindled to ovoid cell and abundant slightly eosinephillic cytoplasm. Small to large multinucleate cells appeared with distinct nucleoli. IDCS had a moderate cytologic atypia and a low mitotic rate. Necrosis was less seen^4^. In our overview, we found that IDCS had epithelioid cells and mononucleated or multinucleated tumor giant cells, a high mitotic rate, diffuse necrosis and granuloma^5, 16, 17^.

Patients usually present with painless lymph node enlargement or extranodal masses. Systemically atypical symptoms include fever, weight loss, fatigue and night sweats. IDCS were often misdiagnosed as carcinoma, melanoma, sarcoma, and so on^18–20^. There was diversity in histopathological morphology. Immunohistochemically, there is no specific marker for IDCS. Therefore, the diagnosis of IDCS was difficult and achieved by excluding other diseases. IDCS cells were consistently positive for S100 protein and vimentin, weakly positive for fascin, CD68, lysozyme and CD45. The tumor cells were negative for CD21, CD23, CD35 and CD1a, CKpan, cam5.2, EMA, HMB45, Melan A, CD3, CD20, Desmin, SMA, and so on. However, in case 2, the tumor cells expressed CKpan focally, which needed further investigation. Ultrastructurally, the cells demonstrated complex features of interdigitating cells. Birbeck granules and melanosomes were not seen from other studies^4, 18^.

Patients have variations in prognosis (some patients received operation and chemotherapy and radiotherapy, but PFS was no more than 1 month, some patient had no treatment after surgery but PFS lasted for years)^15^. The extreme rarity of IDCS impeded researches about the prognostic factors for the diverse prognosis. Pas^21^ *et al*. reported necrosis and mitosis were unconfirmed prognosis factors. Therefore, we proposed a pooled analysis for IDCS for the histopathological prognosticators. Our analysis showed median PFS of diffuse necrosis was 3 months but patients with no necrosis or focal necrosis had not observed median PFS, with a significant difference. Caner Saygin^22^ *et al*. studied 25 cases of IDCS and reported that necrosis was not an indicator of poor prognosis. It was not conflict with this study, because we divided the necrosis into focal necrosis and diffuse necrosis and the diffuse necrosis indicated the poorer prognosis.

Our analysis indicated high mitotic count (≥5/10 HPF) and the nuclear atypia were not associated with the poor prognosis the results were in accord with previous report^21^. We also found median age has not any significant differences between tumor lesion, cellular atypia and necrosis. However, the patients with nuclear mitosis <5/10HPF were younger than the patients with nuclear mitosis ≥5/10HPF. The significance is needed to be confirmed further. Patients with diffused lesion had a shorter PFS. These results indicated diffuse lesion had a worse prognosis and were identical with the reported in literature^5, 23^.

However, there are some limitations of our analysis. Our data stemmed from the published literature and we could not obtain complete clinical, pathological and follow-up data for some patients. It is hard to compare IDCS patients with uniform treatments. Therefore, it was necessary to implement a consolidated and well-designed prospective design in further study.

In conclusion, we found that the diffuse lesion and diffuse necrosis of IDCS was associated with a worse biological behavior and prognosis, and the cellular atypia and mitosis had no distinct relevance with the biological behavior and prognosis of IDCS.
